# Chewing Gum May Alleviate Degree of Thirst in Patients on Hemodialysis

**DOI:** 10.3390/medicina60010002

**Published:** 2023-12-19

**Authors:** Yi-Qi Chen, Chia-Liang Wang, Ai-Hua Chiu, Ming-Chen Yeh, Tsay-I Chiang

**Affiliations:** 1 Department of Nursing, Hungkuang University, Taichung 403, Taiwan; q7834683@yahoo.com.tw (Y.-Q.C.); mcyeh@hk.edu.tw (M.-C.Y.); 2 Department of Nephrology, Kuang-Tien General Hospital, Taichung 433, Taiwanlovereona0403@gmail.com (A.-H.C.); 3 Department of Nutrition, Hungkuang University, Taichung 403, Taiwan

**Keywords:** chewing gum, thirst, hemodialysis

## Abstract

*Background and Objectives*: Fluid restriction commonly causes dry mouth in patients on hemodialysis (HD). The observed effects of chewing gum on the amount of saliva, interdialytic weight gain (IDWG), and degree of thirst in patients on HD have been inconsistent. We investigated whether chewing gum can modulate these three parameters in patients on HD. *Materials and Methods*: We used purposive sampling to enroll 37 patients on HD who were then randomly divided into the chewing gum group (*n* = 19) and the control (non–chewing gum) group (*n* = 18). The degree of thirst was assessed using a self-rated visual analog scale (VAS) and a summated xerostomia inventory (SXI), respectively. *Results*: The degree of thirst after dialysis was significantly lower in the chewing gum group than in the control group. The amount of saliva was higher in the chewing gum group than in the control group before dialysis, but the saliva amount was comparable between the groups after dialysis. Furthermore, the amount of saliva was not associated with IDWG in patients on HD before or after dialysis. *Conclusions*: Chewing gum may alleviate the feeling of thirst but may not affect the amount of saliva and IDWG in patients on HD. Therefore, we suggest that chewing gum may be a useful approach to improve dry mouth and thirst in patients on HD.

## 1. Introduction

Chronic kidney disease (CKD) is a progressive and irreversible degeneration in which the body loses its ability to keep fuel and balance water and electrolytes [1]. Both CKD and its treatment can cause tissue and systemic disorders and directly affect the flow, concentration, and composition of saliva [2]. Kidney failure causes water and sodium excretion [3] and the decrease in urine volume [4]; furthermore, excess fluid intake leads to edema and weight gain in hemodialysis (HD) patients [5]. Most patients on HD must maintain a fluid-restricted diet to prevent fluid overload during dialysis sessions. High fluid intake increases interdialytic weight gain (IDWG), resulting in hypertension, acute pulmonary edema, congestive heart failure, cardiovascular comorbidity, and eventually death. Patients on HD with dry mouth and thirst may have a high fluid intake [6,7]. Some studies have indicated an association between thirst and IDWG in patients on HD, but this finding is not currently supported by other reports [8,9].

Thirst is one of the most important and commonly observed symptoms in patients on HD. In total, 68 to 86 percent of patients on HD express experiencing thirst or dry mouth, which can affect their quality of life and lead to discomfort and distress [10]. In addition, thirst and dry mouth can lead to significant weight gain between two dialyses [11]. There are many ways to treat dry mouth and thirst. Chewing gum has become a viable option due to its stimulation of the salivary glands, its mechanical cleansing of the teeth, and its antimicrobial effects [12]. It also increases the flow of saliva and improves mouth dryness [8].

Fluid intake restriction causes patients on HD to experience dry mouth and increased thirst, necessitating methods to stimulate saliva secretion. A limited number of studies have examined the effect of chewing gum on dry mouth in patients on HD, and the observed effects have been inconsistent. For example, a positive effect of chewing gum was observed in Bots’ study [9], but a null effect was reported by Duruk et al. (2016) [10]. Therefore, the effect of chewing gum on dry mouth in patients on HD remains unclear.

In the present case–control study involving patients on HD, we explored whether chewing gum can alleviate dry mouth and thirst, increase the amount of saliva, and decrease IDWG between dialysis sessions. We also evaluated the association between the amount of saliva with salivary pH and changes in IDWG between dialysis sessions.

## 2. Materials and Methods

### 2.1. Patients and Study Protocol

This study was approved by the Institutional Review Board of Kung Tien General Hospital, Taichung (KTGH No: 11054). We used purposive sampling to enroll 37 patients on HD from the Dialysis Center of Kung Tien General Hospital. This study enrolled individuals aged 20 and above with a minimum six-month history of hemodialysis that consisted of three sessions per week, each lasting four hours. Participants could not be receiving current oxygen therapy, had to be able to chew solid food, exhibit clear consciousness and literacy, and have no visual or hearing impairments. Voluntary participation was subject to signing an informed consent form. Individuals with oral diseases or removable dentures, central nervous system disorders, a history of chemotherapy or radiation therapy, psychiatric disorders, difficulty cooperating with the study, or a known allergy to chewing gum were excluded.

**Figure 1:**
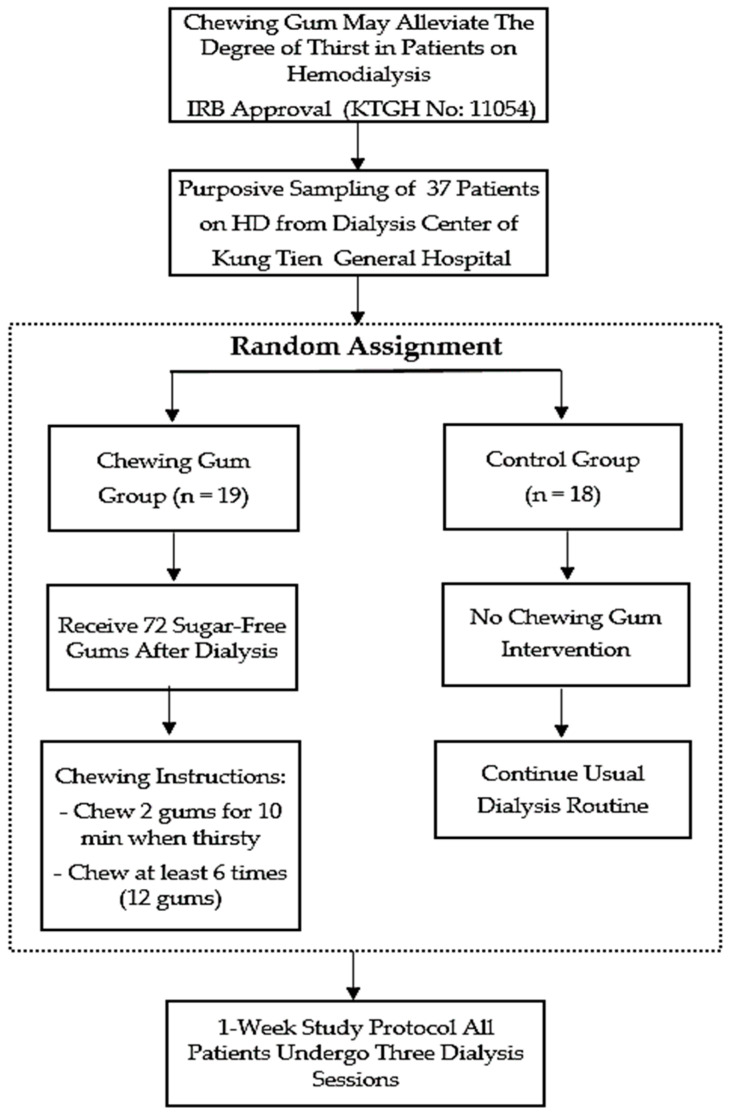
Study protocol.

All participants were randomly divided into the chewing gum group, who received sugar-free gum before or after dialysis (*n* = 19), and the control group, who did not receive chewing gum before or after dialysis (*n* = 18). The study protocol was designed for one week, and all patients underwent three dialysis sessions. The chewing gum group patients received 72 sugar-free gum portions after dialysis for six days’ use. They were instructed to chew two gum portions for 10 min each time they felt thirsty and chew at least six times (12 portions) per day (see Figure 1).

### 2.2. Assessment of Degree of Thirst

Thirst was assessed using a 100 mm self-rated visual analog scale (VAS) [9] and the Summated Xerostomia Inventory (SXI) [8]. The VAS was defined as the negative and the positive on the left and right, respectively. The SXI is a validated questionnaire comprising 11 items, each of which is rated on a five-point Likert scale. Both tools were used with all patients before and after a dialysis session.

### 2.3. Analysis of the Amount of Saliva and Its pH Value

The amount of saliva in patients before and after a dialysis session was assessed using the Schirmer tear test, according to a previous report [13]. The tear test filter paper was placed on the floor of the patients’ mouth for 3 min, following which color developed on the paper. If the length of the colored portion was <25 mm after 3 min, inadequate saliva secretion was recorded.

Salivary pH was measured using acid-base test strips (Toyo, Advantec, CT-NO20), which have 16 pH values as follows: 5.0, 5.2, 5.4, 5.6, 5.8, 6.0, 6.2, 6.4, 6.6, 6.8, 7.0, 7.2, 7.4, 7.6, 7.8, and 8.0 [10]. The strips were placed on the floor of the participant’s mouth when the tongue was attached to jaws, and the pH value was determined from the color of the strips. Red to yellow indicated acidic pH, and yellow to green indicated basic pH.

### 2.4. Assessment of IDWG and Blood Pressure

Each participants’ body weight was measured before and after each HD session. IDWG was defined as the amount of fluid (kg) removed during the HD session. To better assess the changes in IDWG, the ultrafiltration rate was set according to the IDWG in each HD session and correlated with the participant’s post-dialysis body weight against the target dry weight. The participants’ systolic blood pressure (SBP) and diastolic blood pressure (DBP) were recorded using a sphygmomanometer.

### 2.5. Statistical Analysis

All statistical analyses were conducted using SPSS v25.0 (Statistical Package for the Social Sciences), and a two-tailed *p* value of 0.05 was deemed significant. Data are expressed as mean ± standard deviation. The Spearman correlation coefficient was used to assess the correlation between variables.

## 3. Results

### 3.1. Patient Demographics

We enrolled 37 HD patients. After being provided with explanations of the study aims and procedures, all patients provided informed consent before the study commenced. The patients’ clinicodemographic data are presented in Table 1. The control group patients were non-significantly older than the chewing gum group patients (71.33 ± 8.81 vs. 65.05 ± 11.21, *p* = 0.067; Table 1). No other parameters, including sex, education level, diet habits, chronic diseases, or dialysis year, were significantly different between the two groups.

### 3.2. Comparison of the Amount of Saliva, Salivary pH, Blood Pressure, Body Weight, Water Loss, IDWG, and Degree of Thirst between the Chewing Gum Group and the Control Group

We examined whether the amount of saliva, salivary pH, SBP, DBP, body weight, water loss, IDWG, and thirst differed between the chewing gum and control groups before the intervention of chewing gum. As presented in Table 2, the amount of saliva was significantly higher in the chewing gum group than in the control group before dialysis (16.00 ± 7.92 vs. 9.94 ± 5.08, *p* = 0.009) but not after dialysis (16.08 ± 8.36 vs. 12.27 ± 5.23, *p* = 0.107; Table 2). No between-group differences were noted in terms of salivary pH, SBP, DBP, or body weight before and after dialysis (Table 2). The IDWG level was higher in the chewing gum group than in the control group after dialysis (2.22 ± 0.73 vs. 1.74 ± 0.54, *p* = 0.031, Table 2), but the degree of thirst evaluated using the VAS or SXI did not differ between the groups after dialysis. In the non-chewing gum intervention, IDWG was expectedly higher in the chewing gum group than in the control group after dialysis, but the amount of saliva was unexpectedly greater in the chewing gum group than in the control group before dialysis.

### 3.3. Effect of Chewing Gum on the Amount of Saliva, Salivary pH, CBP, DBP, Body Weight, Water Loss, IDWG, and Thirst before and after HD

The amount of saliva decreased in the chewing gum group after dialysis (12.47 ± 6.27 vs. 16.08 ± 8.36, *p* = 0.057, Table 3). Chewing gum did not change the saliva pH value, CBP, DBP, body weight, or water loss before and after dialysis. Notably, the degree of thirst evaluated using the VAS and SXI decreased significantly following the chewing gum intervention in patients on HD after dialysis compared to those without the chewing gum intervention (VAS: 5.16 ± 1.64 vs. 7.37 ± 2.34, *p* < 0.0001, t = 4.483; SXI: 13.89 ± 3.16 vs. 16.79 ± 3.19, *p* = 0.004, t = 3.334; Table 3). These results suggest that chewing gum may improve the feeling of thirst but not change other physiological parameters.

### 3.4. Correlation between the Amount of Saliva and Salivary pH in the Chewing Gum Group before and after Dialysis

**Figure 2:**
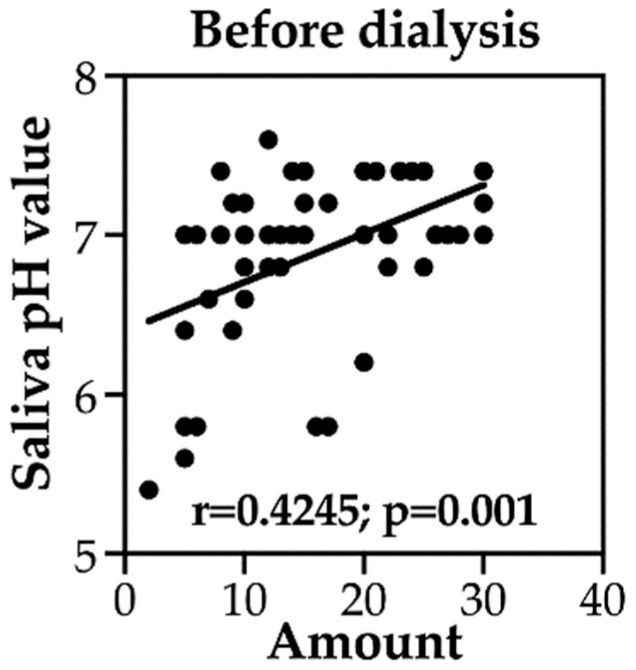
Correlation of the amount of saliva with salivary pH in the chewing gum group of patients on HD before dialysis.

**Figure 3:**
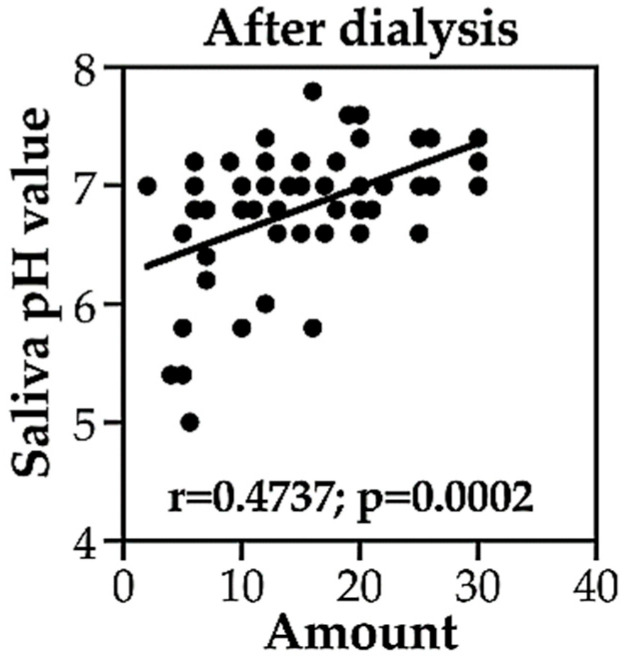
Correlation of the amount of saliva with salivary pH in the chewing gum group of patients on HD after dialysis.

We examined the correlation between the amount of saliva and salivary pH in the chewing gum group before and after dialysis. Both parameters were measured before and after each of the three dialysis sessions. As presented in Table 4 and Figure 2 and Figure 3, the amount of saliva was associated with salivary pH in patients on HD before the second (r = 0.595, *p* < 0.01) and third dialysis sessions (r = 0.481, *p* < 0.05) but not before the first dialysis session. The amount of saliva correlated with salivary pH after the first and second dialysis sessions (r = 0.494 and 0.513, respectively, both *p* < 0.05) but not the third dialysis session. These results suggest that the increase in saliva following chewing gum may be associated with more acidic salivary pH in patients with HD.

## 4. Discussion

In the general population, the rate of saliva secretion averages 0.3 to 0.5 mL/min [14,15]. An unstimulated salivary flow rate of at least 0.1 to 0.3 mL/min is thought to be needed to avoid the development of dry mouth or xerostomia [15]. Salivary flow is significantly decreased in patients receiving HD, typically by 20 to 55 percent [16,17]. The reduced salivary flow observed in HD patients may be caused by salivary gland fibrosis and atrophy, medication use, fluid intake restriction, and effects of dialysis procedures [18].

There are many ways to treat dry mouth and thirst. For example, to relieve the complications and discomfort caused by dry mouth, patients can sip on water throughout the day or place ice in the mouth to intake fluids slowly. Other treatments include the use of artificial saliva; the administration of parasympathetic mimicking drugs, such as pilocarpine hydrochloride; mouthwash; and non-alcoholic drinks [19]. Among these, chewing gum has become increasingly important due to its stimulation of salivary glands, the mechanical cleansing of the teeth, and its antimicrobial effects. It also increases the flow of saliva and improves mouth dryness [18,20,21,22].

In the present study, each patient’s degree of thirst was evaluated using VAS and SXI. Thirst significantly decreased in the chewing gum group compared with the control group after dialysis (Table 3). At baseline, the amount of saliva was higher in the chewing gum group than in the control group before dialysis (Table 2), but this difference was no longer significant after dialysis (Table 3). In addition, the amount of saliva was not associated with IDWG in patients on HD before and after dialysis (Supplementary Tables S1 and S2). Our results are consistent with previous reports indicating that chewing gum may alleviate feelings of thirst but not modulate the amount of saliva and IDWG in patients on HD [9,23]. However, the present results do not follow those of an integrative review of the effect of chewing gum on thirst, which indicated that chewing gum can increase the amount of saliva and reduce the feeling of thirst in patients on HD [24,25]. Other studies associated IDWG in patients on HD with dry mouth and thirst [4,9]. These results differ from our research findings.

Thirst is a subjective sensation in long-term HD patients, activated by a reduction in extracellular fluid and an increase in plasma osmotic pressure [6,7]. Typically, thirst symptoms in dialysis patients commence four to six hours after the completion of dialysis, persisting between two dialysis sessions, with only partial relief during the dialysis process [19]. In our study, the patients’ blood sodium concentrations were 135.2 ± 2.83 in the experimental group and 134.27 ± 3.33 in the control group.

A large-scale clinical crossover study (*n* = 65) concluded that a two-week chewing gum intervention significantly reduced the degree of thirst in patients on HD [9]. Similar findings were reported by two other studies that consisted of 42 and 60 patients on HD, respectively [8,20]. However, other studies have reported no effect of chewing gum on thirst and dry mouth in patients on HD [5,7]. Even though the present study included a relatively small sample of patients on HD (*n* = 37), our results revealed that chewing gum can alleviate the feeling of thirst. In addition, changes in blood pressure, weight, dialysis dehydration volume, IDWG, and chronic conditions such as hypertension and diabetes, before and after hemodialysis, did not show significant predictive power for the factor of thirst discomfort.

Although chewing gum did not influence the amount of saliva and salivary pH (Table 3), the amount of saliva was positively correlated with salivary pH in patients before and after dialysis (Table 4). Furthermore, when assessing the immediate effects of chewing gum in the experimental group, saliva volume increased from 16.00 ± 7.92 to 27.11 ± 6.66, and the pH value increased from 6.76 ± 0.52 to 7.244 ± 0.29. The subjects reported temporary relief in their self-perceived degree of thirst. A previous report indicated that chewing bicarbonate-containing sugar-free gum and standard sugar-free gum did not influence salivary pH value from 0 to 20 min [26]. To the best of our knowledge, ours is the first study to report the association between the amount of saliva and salivary pH in patients on HD.

The study’s potential limitation is its small sample size (*n* = 19) and control group (*n* = 17). This small sample size was due to the occurrence of the COVID-19 pandemic during the enrollment period for the study. Pandemic restrictions resulted in a constrained sample size due to considerations for exposure and infection risks. This limitation may impact the generalizability of the study findings to a larger population. To strengthen the reliability of our conclusions, we are actively exploring future research directions that involve larger and more diverse populations.

## 5. Conclusions

Our data indicate that chewing gum several times a day for one week can alleviate the degree of thirst in patients on HD, but it does not affect the amount of saliva and IDWG. We therefore suggest that patients on HD be encouraged to chew sugar-free gum to alleviate their complaints of thirst and dry mouth, thus improving their quality of life. In summary, chewing sugar-free gum aids in maintaining fresh breath, stimulating saliva production, and alleviating thirst. It may enhance attention and alertness, and it is considered to contribute to stress relief. The findings of this study can be applied to populations of clinical HD patients experiencing dry mouth, as well as to patients who undergo prolonged fasting. This applicability is attributed to the fact that the intervention does not alter any physiological parameters in the bloodstream. However, caution is advised against excessive chewing, as it may exert pressure on the temporomandibular joint, and certain individuals may experience allergic reactions to gum ingredients. For individuals with specific health conditions, seeking counsel from a medical professional beforehand is advisable.

## Figures and Tables

**Table 1:** Clinicodemographic characteristics of the chewing gum and control groups.

Parameter	Patient No. (%)	Study Group	Control Group	*p* Value
		*n* = 19 (%)	*n* = 18 (%)	
**Sex**				
Male	18 (48.6)	9 (47.4)	9 (50.0)	
Female	19 (51.4)	10 (52.6)	9 (50.0)	0.873
**Education level**				
None	6 (16.2)	2 (10.5)	4 (22.2)	
Elementary	18 (48.6)	8 (42.1)	10 (55.6)	
Junior high	9 (24.3)	8 (42.1)	1 (5.6)	
Senior high	3 (8.1)	1 (5.3)	2 (11.1)	
College/University	1 (2.7)	0 (0.0)	1 (5.6)	0.105
**Dietary habit**				
Light taste	13 (35.1)	6 (31.6)	7 (38.9)	
General taste	17 (45.9)	9 (47.4)	8 (44.4)	
Heavy taste	7 (18.9)	4 (21.1)	3 (16.7)	0.882
**Chronic disease**				
Hypertesion	24 (64.9)	13 (68.4)	11 (61.1)	0.642
Diabetes	23 (62.2)	12 (63.2)	11 (61.1)	0.898
Hyperlipidemia	1 (2.7)	1 (5.3)	0 (0.0)	0.324
Other	5 (13.5)	2 (10.5)	3 (16.7)	0.585
**Age (years)**	68.11 ± 10.47	65.05 ± 11.21	71.33 ± 8.81	0.067
**Dialysis year**	6.17 ± 6.15	7.50 ± 7.62	4.77 ± 3.79	0.176

**Table 2:** Amount of saliva, salivary pH, blood pressure, body weight, water loss, IDWG, and thirst in patients on HD without the intervention of chewing gum.

Parameter	Before Dialysis	After Dialysis
**Amount of saliva**		
Study	16.00 ± 7.92	16.08 ± 8.36
Control	9.94 ± 5.08	12.27 ± 5.23
P (t)	0.009 (2.750)	0.107
**Salivary pH**		
Study	6.76 ± 0.52	6.67 ± 0.65
Control	6.68 ± 0.61	6.60 ± 0.50
*p* value	0.671	0.704
**CBP**		
Study	142.58 ± 22.10	133.53 ± 22.64
Control	146.94 ± 23.51	136.06 ± 25.53
p value	0.564	0.751
**DBP**		
Study	70.58 ± 8.54	70.00 ± 10.51
Control	75.61 ± 12.31	71.39 ± 12.46
*p* value	0.564	0.761
**Body weight**		
Study	65.73 ± 10.16	63.51 ± 9.91
Control	65.47 ± 11.98	63.53 ± 12.03
*p* value	0.944	0.996
**Water loss**		
Study	-	2.21 ± 0.65
Control	-	1.89 ± 0.75
*p* value	-	0.184
**IDWG**		
Study	-	2.22 ± 0.73
Control	-	1.74 ± 0.54
P (t)	-	0.031 (2.243)
**Thirst (VAS)**		
Study	-	7.37 ± 2.34
Control	-	7.17 ± 1.62
*p* value	-	0.763
**Thirst (SXI)**		
Study	-	16.79 ± 3.19
Control	-	16.89 ± 4.71
*p* value	-	0.941

An independent *t*-test was used to calculate the *p* and t values.

**Table 3:** Effect of chewing gum on the amount of saliva, salivary pH, CBP, DBP, body weight, water loss, IDWG, and thirst in patients on HD before and after HD.

Parameter	Control Group	Study Group	P (t)
**Amount of saliva**			
Before dialysis	16.00 ± 7.92	14.21 ± 6.66	0.395
After dialysis	16.08 ± 8.36	12.47 ± 6.27	0.057
**Salivary pH**			
Before dialysis	6.76 ± 0.52	6.88 ± 0.59	0.396
After dialysis	6.67 ± 0.65	6.75 ± 0.55	0.663
**CBP**			
Before dialysis	142.58 ± 22.10	137.58 ± 41.14	0.492
After dialysis	133.53 ± 22.64	129.42 ± 17.10	0.461
**DBP**			
Before dialysis	70.58 ± 8.54	74.32 ± 11.95	0.196
After dialysis	70.00 ± 10.51	72.26 ± 12.10	0.409
**Body weight**			
Before dialysis	65.73 ± 10.16	65.83 ± 10.55	0.648
After dialysis	63.51 ± 9.91	63.55 ± 10.18	0.758
**Water loss**			
After dialysis	2.21 ± 0.65	2.26 ± 0.70	0.765
**IDWG**	2.22 ± 0.73	2.33 ± 0.82	0.579
**Thirst (VAS)**	7.37 ± 2.34	5.16 ± 1.64	<0.0001 (4.483)
**Thirst (SXI)**	16.79 ± 3.19	13.89 ± 3.16	0.004 (3.334)

An independent *t*-test was used to calculate the *p* and t values.

**Table 4:** Correlation of the amount of saliva with salivary pH in the chewing gum group of patients on HD before and after dialysis.

**Saliva Amount**	**Saliva pH 1**	**Saliva pH 2**	**Saliva pH 3**
**Before dialysis**			
Amount 1	0.263		
Amount 2		0.595 **	
Amount 3			0.481 *
**After dialysis**			
Amount 1	0.494 *		
Amount 2		0.513 *	
Amount 3			0.453

* *p* < 0.05 ** *p* < 0.01 Spearman Rank Correlation Analysis.

## Data Availability

The data that support the findings of this study are available from the corresponding author, T.-I.C., upon reasonable request.

## Supplementary Materials

The following supporting information can be downloaded at: https://www.mdpi.com/article/10.3390/medicina60010002/s1, Table S1: Correlation between salivary volume before & after dialysis and Interdialytic Weight Gain (IDWD); Table S2: Correlation between Salivary Volume after Dialysis and Interdialytic Weight Gain (IDWD).
