# Supplementary material for: Chewing Gum May Alleviate Degree of Thirst in Patients on Hemodialysis
Source: Medicina (Kaunas). 2023 Dec 19;60(1):2. doi: 10.3390/medicina60010002 (PMC10817533; doi:10.3390/medicina60010002)
Supplement: Supplementary file 1 [file medicina-60-00002-s001.zip › medicina-2736723-supplementary.pdf]

Suppleme Table S1

Correlation Between Salivary Volume Before Dialysis and Interdialytic Weight Gain (IDWD)

	IDWD (first)	IDWD (second)	IDWD (third)
Salivary Volume (first)	-0.233		
Salivary Volume (second)		0.104	
Salivary Volume (third)			0.287

*p<0.05，**p<0.01

Suppleme Table S2

Correlation Between Salivary Volume After Dialysis and Interdialytic Weight Gain (IDWD)

	IDWD (first)	IDWD (second)	IDWD (third)
Salivary Volume (first)	-0.222		
Salivary Volume (second)		0.033	
Salivary Volume (third)			0.437

*p<0.05，**p<0.01
